# Conductive Fibers of Chitosan/DNA Interfacial Polyelectrolyte Complexation Incorporating Carbon Nanotubes

**DOI:** 10.1021/acsami.6c00347

**Published:** 2026-04-08

**Authors:** Yoshinobu Utagawa, Masahiro Takinoue, Shin-ichiro M. Nomura, Yusuke Sato, Hiroaki Onoe, Toshinori Fujie, Hikaru Nakazawa, Mitsuo Umetsu, Hiroya Abe, Hitoshi Shiku, Kosuke Ino

**Affiliations:** † Graduate School of Engineering, 13101 Tohoku University, Sendai 980-8579, Japan; § School of Life Science and Technology, Institute of Science Tokyo, Yokohama 226-8501, Japan; ∥ Research Center for Autonomous Systems Materialogy, Institute of Integrated Research, Institute of Science Tokyo, 4259 Nagatsuta-cho, Midori-ku, Yokohama 226-8501, Japan; ⊥ Department of Bioscience and Bioinformatics, 12924 Kyushu Institute of Technology, 680-4 Kawazu, Iizuka, Fukuoka 820-8502, Japan; # Department of Mechanical Engineering, Keio University, Hiyoshi, Kohoku-ku, Yokohama 223-8522, Japan; 7 Frontier Research Institute for Interdisciplinary Sciences, Tohoku University, Sendai 980-8578, Japan; 8 Laboratory for Chemistry and Life Science, Institute of Integrated Research, Institute of Science Tokyo, Yokohama 226-8501, Japan; 9 Department of Life Science and Technology, Institute of Science Tokyo, Yokohama 226-8501, Japan; 10 Institute of Biomedical Engineering, Institute of Science Tokyo 4259 Nagatsuta-cho, Midori-ku, Yokohama 226-8501, Japan

**Keywords:** conductive fiber, interfacial polyelectrolyte complexation, chitosan, DNA, carbon nanotube

## Abstract

Conductive fibers hold considerable potential in wearable electronics, soft robotics, and flexible sensing platforms. However, conventional fabrication typically relies on specialized instrumentation and high-temperature processing, limiting accessibility and sustainability. Herein, we present conductive fibers composed of chitosan and DNA–carbon nanotubes (CNTs) prepared via interfacial polyelectrolyte complexation (IPC). This simple, low-energy method requires neither complex instrumentation nor thermal treatment. The resulting IPC fibers exhibited stable electrical conductivity, which was attributed to interactions between DNA and CNTs. Notably, the conductive fibers demonstrated self-healing capability, wherein severed segments rejoined upon hydration with restoration of conductivity. In addition, the fiber demonstrated conductivity and stretchability in the wet state, enabling the monitoring of strain-induced current changes for motion tracking capture. Furthermore, Janus fibers were fabricated by aligning the fibers with magnetic beads in parallel, yielding conductive/magnetic hybrids that demonstrated electrical switching under remote magnetic actuation. Collectively, these findings highlight a scalable and sustainable strategy for fabricating reconfigurable conductive fibers for biointerfaced and flexible electronics.

## Graphical abstract

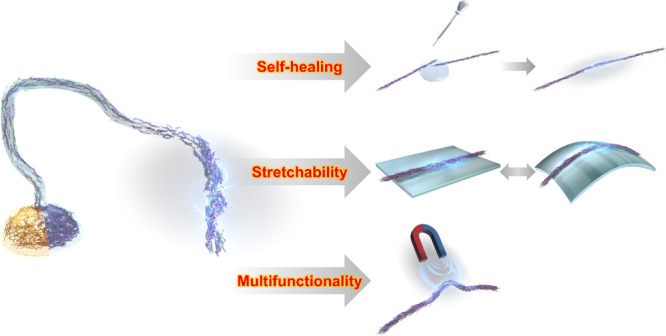

## Introduction

Fibers are ubiquitous materials that play a crucial role in industrial and biomedical fields. In particular, conductive fibers have gained significant attention because of their potential applications in wearable electronics,^1^ flexible electronics,^2^ and smart textiles.^3^ Various fabrication methods have been explored to develop conductive fibers, including electrospinning,^4^ wet spinning,^5^ carbonization,^6^ adaptable covalently cross-linked approaches^7^ thermally drawn polymer approaches,^8^ freeze printing,^9^ and metal coating techniques.^10^ However, many of these approaches require sophisticated equipment, high processing temperatures, or lengthy multistep procedures, limiting their scalability and practical accessibility.

Polymer complex fibers have attracted attention because of their excellent abilities, including reversibility, stimuli responsiveness, and self-healing.^11^ Among various fabrication strategies, interfacial drawing enables fiber fabrication under simpler and milder conditions by drawing an interfacial polyelectrolyte complexation (IPC) film formed between droplets of oppositely charged polymer solutions.^12^ This approach is considerably simpler than conventional fiber fabrication methods because it does not require complex processes or specialized equipment. Various polyelectrolyte pairs have been explored, including chitin/alginate,^13^ chitosan/alginate,^14^ and chitosan/DNA.^15^ Owing to their formation at neutral pH and room temperature using only aqueous solutions, IPC fibers have been used in cell culture studies.^12–15^ Beyond simple structures, more complex fiber structures, such as multiple,^13^ core–shell,^15^ and hollow fibers,^16^ have also been demonstrated. In addition to cell cultures, IPC fibers have been used in diverse functional applications. For instance, fibers incorporating reduced graphene oxide have been reported as fiber-shaped electrodes.^17^ In addition, interfacial nanoparticle complexation (INC) filaments prepared using a similar method exhibited high conductivity by incorporating carbon nanotubes (CNTs).^18,19^ Conductive fibers must possess various functions in addition to conductivity for practical applications. For instance, self-healing function prevents deterioration caused by mechanical stimuli and enables long-term use.^20^ In addition, flexibility enables real-time monitoring of motion signals through strain sensing.^21^ However, thus far, conductive IPC and INC fibers have mainly been evaluated for electrochemical and electrical properties, whereas their potential in self-healing, strain sensing, and the fabrication of multifunctional fibers for electronic devices remain largely unexplored.

In this study, we developed conductive fibers using IPC techniques with chitosan and DNA–CNT composites and demonstrated their self-healing, strain-sensing, and multifunctional capabilities. DNA offers several advantages, including natural polymer properties, biocompatibility, molecular recognition capability, chemical modifiability, and information carrying capability. In addition, DNA can be used as a bioprogramming platform to design DNA sequences.^22^ CNTs are well dispersed in a DNA solution because of π–π interactions between DNA and CNTs.^23^ DNA–CNT composite fibers have been used in bioinks for 3D printing.^24^ Beyond this application, the simple fabrication of fibers via electrostatic interactions with chitosan enables easy processing, while the intrinsic antibacterial properties of chitosan highlight its potential for future applications in cell culture and biodevices.^25^ Moreover, conductive gel fibers based on DNA–CNT are fabricated by wet spinning, where chitosan serves as a coagulant and electrostatic interactions between polymers govern fiber formation.^26^ In contrast, the fibers presented in this study are formed by interfacial drawing between chitosan and DNA–CNT, resulting in a fundamentally different fiber architecture. Because DNA is an essential structural component of the IPC fibers, CNTs can be incorporated directly into the fiber through DNA–CNT composites, enabling the formation of homogeneous conductive pathways in the fibers. As a result, conductive IPC fibers exhibiting multifunctional properties, including self-healing and strain-sensing capabilities, can be realized. Initially, the properties of chitosan/DNA–CNT fibers were characterized and compared with those of chitosan/alginate–CNT fibers because alginate does not interact with CNTs. Conductivity was then evaluated by varying the concentrations of both DNA and CNTs. The fibers further demonstrated self-healing, strain-sensing, and the potential for multifunctional fibers. The experimental demonstration of these functionalities represents an advance beyond previously reported conductive IPC or INC fibers. Overall, these features highlight the considerable potential of chitosan/DNA–CNT fibers for future applications in biointerfaced and flexible electronics.

## Materials and Methods

### Preparation of CNT Dispersions and Evaluation of Dispersibility

A 1.0% (w/v) double-stranded DNA from salmon milt (Tokyo Chemical Industry Co., Ltd., Japan) was dissolved in Milli-Q water. A 1.0% (w/v) sodium alginate (FUJIFILM Wako Pure Chemicals Corporation, Japan) was dissolved in Milli-Q water, and the solution was used as a control. Multiwalled CNTs (20–30 nm; FUJIFILM Wako Pure Chemicals Corporation) were dispersed in DNA or alginate solution by sonication for 10 min in an ultrasonic bath (200 W; US-105; SND Co., Ltd., Japan).

The CNT dispersion was centrifuged at 2500 × g for 30 min, and supernatants were obtained. The supernatants were analyzed using UV–vis spectroscopy. In addition, zeta potentials and particle sizes were analyzed using Zetasizer Ultra (Malvern Panalytical, UK).

### Fabrication of IPC Fibers

A 0.25% (w/v) chitosan (Sigma-Aldrich, USA) was dissolved in an acetate solution (pH 5.6). Then, 100 μL droplets of chitosan solution and CNT dispersed DNA solution were prepared on a hydrophobic film. IPC fibers were formed by manually drawing the droplet interface using tweezers. The fibers were dried at room temperature for 1–2 h while suspended in air using a dish. The dried fibers were analyzed by scanning electron microscopy (SEM; S-4800; Hitachi High-Tech Corporation, Japan).

### Conductivity Measurements

Glass plates (Matsunami Glass Ind., Ltd., Japan) were sputtered with Au. The IPC fibers were fabricated and placed on glass plates between Au. After drying the IPC fibers, the glass plates with Au were connected to a potentiostat (HA1010 mM4; Hokuto Denko, Japan). Subsequently, cyclic voltammetry was performed. The resistances of the fibers were calculated from the slopes of the cyclic voltammograms. In addition, the average cross-sectional area was calculated using an average of 30 locations within the image. The conductivity was calculated using the resistance values, cross-sectional areas, and the fiber length (10 mm). The number of independent samples for each condition was determined based on the variability in diameter and conductivity measurements, particularly near the percolation threshold where larger fluctuations were observed.

After cutting the fibers, both ends were wet with Milli-Q water and dried at room temperature. The conductivity of the repaired fibers was monitored.

### DNase Treatments

A Tris-HCl buffer (pH 8) containing 0.1 U/μL DNase and 0.5 mM MgSO_4_ was prepared. Before the treatment, the conductivity of the fibers was measured. Post treatment, 10 μL of the solution was dropped on the center of the fibers, and the fibers were left to stand at 37 °C for 30 min. The treated fibers were washed with Milli-Q water. After drying at room temperature, the conductivity of the fibers was measured again.

### Temperature Treatments

The fabricated IPC fibers were placed on Au-sputtered glass plates and sealed with polyimide tape. Subsequently, the fibers were attached to a hot plate. Mineral oil was applied to the attached fibers to prevent drying. The conductivity of the fibers was monitored by increasing the temperature from room temperature to 100 °C. The display temperature of the hot plate reached 100 °C in approximately 2 min.

### Conductive/Magnetic Janus Fibers

DNA was dissolved in a suspension of magnetic beads (Dynabead; diameter = 1.0 μm; Thermo Fisher Scientific). Conductive fibers composed of 0.25% (w/v) chitosan and 1% (w/v) DNA containing 50 mg/mL CNTs and magnetic fibers composed of 0.25% (w/v) chitosan and 1% (w/v) DNA containing magnetic beads were simultaneously formed. The fibers were integrated, and the integrated fibers were dried at room temperature.

## Results and Discussion

### CNT Dispersion in a DNA Solution

Before fiber fabrication, CNT dispersion was investigated using milli-Q water, DNA solution, and alginate solution. After 24 h from sonication, CNTs were well dispersed in DNA and alginate solutions (Figure S1A). However, the contribution of intermolecular interactions and viscosity to dispersibility cannot be independently determined. Then, supernatants after centrifugation were observed. The supernatant of the DNA solution became intensely black, confirming that the CNTs were well dispersed (Figure S1B). In addition, absorbance was measured via UV–vis spectroscopy. Both DNA solutions and DNA–CNT suspensions showed absorbance peak at 260 nm (Figure S1C). The dispersion of CNTs resulted in an overall increase in absorbance. Subsequently, zeta potentials were measured using the diluted supernatants (Figure S1D). The zeta potential of CNTs was – 5.97 mV. In contrast, DNA–CNT and alginate–CNT showed – 72.4 and – 80.2 mV, respectively; however, this reflected the negative charge of the polymers in the solution. The zeta potential of the DNA–CNT composites was more negative than that of DNA alone (−37.7 mV), suggesting that a large number of DNA molecules are densely adsorbed onto the surface of a single CNT. Finally, particle size analysis was performed to provide a more direct evaluation of the dispersion state (Figure S1E). A broad distribution was observed for the CNTs, suggesting significant variability in the extent of aggregation. The DNA–CNT composites showed a peak at approximately 200 nm, indicating that aggregation was effectively suppressed. Even after centrifugation, some large aggregates are expected to remain in the Alginate–CNT solution due to its high viscosity. These results suggest that CNTs are well-dispersed in the DNA solution, which can be attributed to the strong interaction between DNA and CNTs.

### Fabrication and Characterization of Chitosan/DNA–CNT Fibers

Figure 1A illustrates the fabrication process. Initially, DNA–CNT composites were fabricated through sonication, enabling high dispersion of CNTs. Subsequently, droplets of the chitosan and DNA–CNT composite solutions were prepared on a hydrophobic film. IPC fibers were formed by drawing the interface of the droplets (Figure 1B). The length of the IPC fibers without CNTs was approximately 1 m (Figure S2). In the latter part of the fiber fabrication process, the fibers gradually became thinner, indicating depletion of the polymer. In contrast, the length of the IPC fibers containing 50 mg/mL CNTs was approximately 0.5 m (Figure S2). At this point, the chitosan solution was depleted, which may be attributed to the increased demand for chitosan by the formation of DNA–CNT composites. The fibers were sufficiently flexible to be tied and twisted (Figures 1C and D). In addition, a mesh was fabricated using these fibers because they adhered to each other when dried (Figure 1E). The mesh size was approximately 2–4 mm; the mesh could support the weight of a coin (Figure S3). We demonstrated that a two-dimensional structure can be fabricated using this fiber. Moreover, the structure of the fibers was maintained for at least 72 h when stored in water (Figure S4).

**Figure 1:**
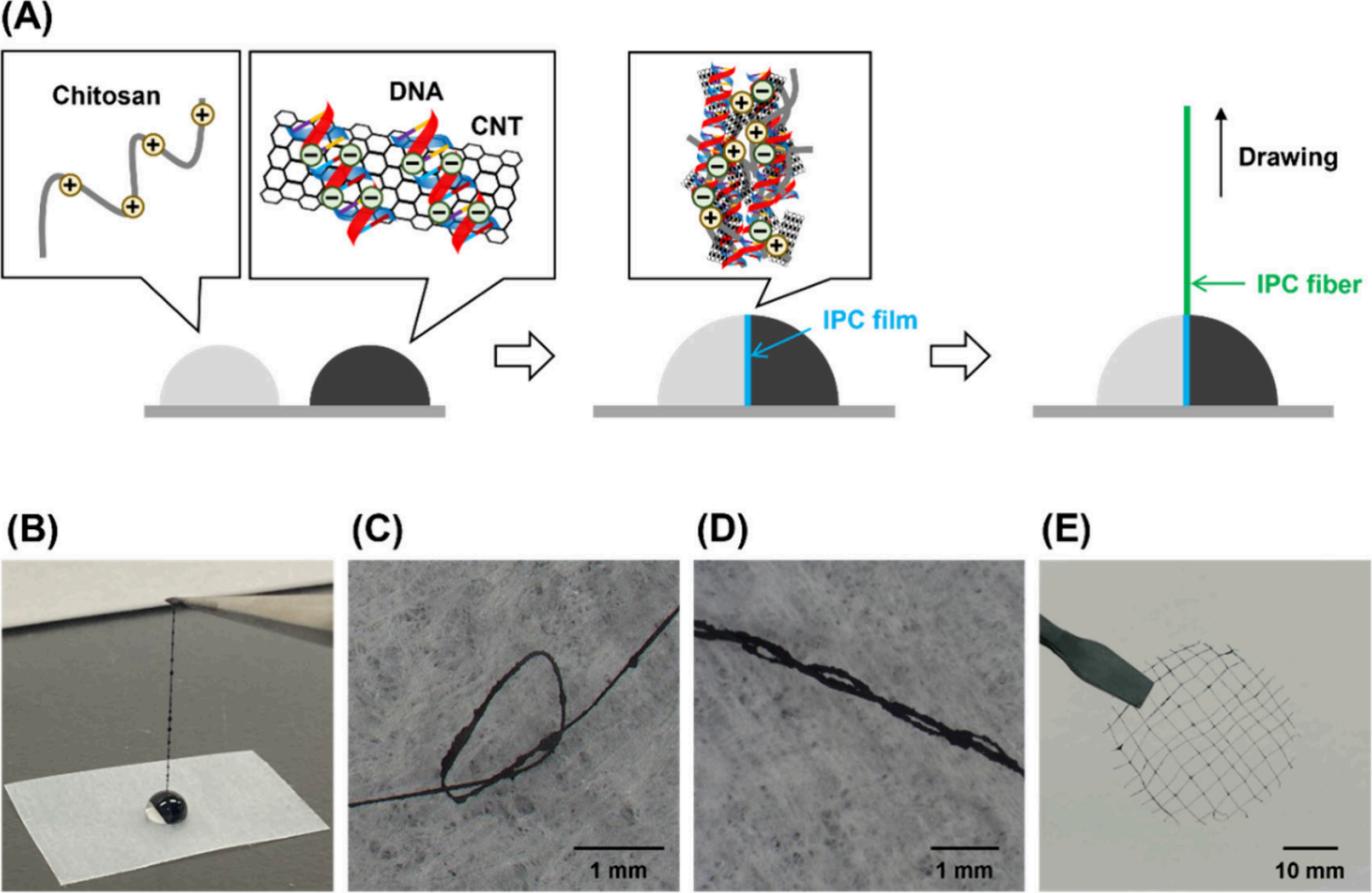
Conductive fibers of chitosan/DNA incorporating CNTs. (A) Schematic illustration of the fabrication process. Two droplets of chitosan and DNA–CNT solutions are merged to form an IPC film within the merged droplet. The IPC fiber is then prepared by drawing the film up. (B) Photograph of the fiber during the process. Photographs of (C) tied, (D) twisted, and (E) cross-aligned chitosan/DNA–CNT fibers. These fibers were dried. (C) A single fiber was tied. (D) Two fibers were twisted. (E) Fibers were aligned before being dried to form a mesh. All fibers were formed with 0.25% (w/v) chitosan, 1% (w/v) DNA, and 50 mg/mL CNT.

SEM images of chitosan/DNA–CNT fibers at different DNA concentrations are shown in Figures 2A–C, whereas Figures 2D and E show the SEM images of chitosan/alginate–CNT fibers with varying alginate concentrations. Rough fibers were formed while using a 0.5% (w/v) DNA solution (Figure 2A, left). This was attributed to low DNA content and excess free CNTs. Consequently, CNT aggregates were incorporated into the fibers. In contrast, smooth fibers were formed in the 5% (w/v) DNA solution, suggesting that the increased proportion of DNA led to a reduced number of unbound CNTs (Figure 2C, left). In contrast to chitosan/DNA–CNT fibers, chitosan/alginate–CNT fibers showed a clear phase separation between chitosan/alginate and the CNTs (Figures 2D and E, left). The surface of the chitosan/DNA was covered with CNTs when using 0.5 or 1% (w/v) DNA solution (Figures 2A and B, middle). In contrast, the surface of the chitosan/alginate composite was smooth (Figures 2D and E, middle), indicating that alginate did not interact with the CNTs. Therefore, chitosan/DNA–CNT fibers may offer superior conductivity and mechanical properties compared to chitosan/alginate–CNT fibers owing to the homogeneous incorporation of CNTs. The cross-sectional structure of the chitosan/DNA–CNT fibers was densely packed (Figures 2A–C, right). In contrast, the interiors of the chitosan/alginate–CNT fibers were less densely packed and showed significant voids (Figures 2D and E, right). Based on fiber weight and volume, the packing densities for chitosan/DNA–CNT and chitosan/alginate–CNT fibers were calculated to be 0.44 and 0.28, respectively, assuming the entire fiber is composed of CNTs due to their dominance in the fiber content. Meanwhile, the distribution of CNTs in both fibers was observed to be uniform. The higher packing density in the chitosan/DNA–CNT fibers may promote more frequent CNT–CNT contacts and facilitate the formation of conductive pathways. In addition, the mechanical properties may also be improved; however, they were not systematically evaluated in this study because the primary focus was on electrical functionality. Therefore, utilizing DNA–CNT composites enabled the fabrication of fibers with characteristic architectures, indicating that the interaction between CNTs and DNA plays a crucial role in structural formation. Moreover, Raman spectroscopy was performed to confirm the interaction between DNA and CNTs. Figure S5A shows Raman spectra of CNTs, DNA–CNT, and alginate–CNT. The G bands of CNTs were observed at approximately 1574 cm^–1^. Both G bands of DNA–CNT and alginate–CNT were shifted to approximately 1580 cm^–1^ (Figure S5B), indicating not only the interaction between DNA and CNT but also physical compression by hydrogel. Then, I_D_/I_G_ ratio was calculated. The I_D_/I_G_ ratio of DNA–CNT was smaller than that of CNTs and alginate–CNT (Figure S5C), indicating the interaction between DNA and CNT.

**Figure 2:**
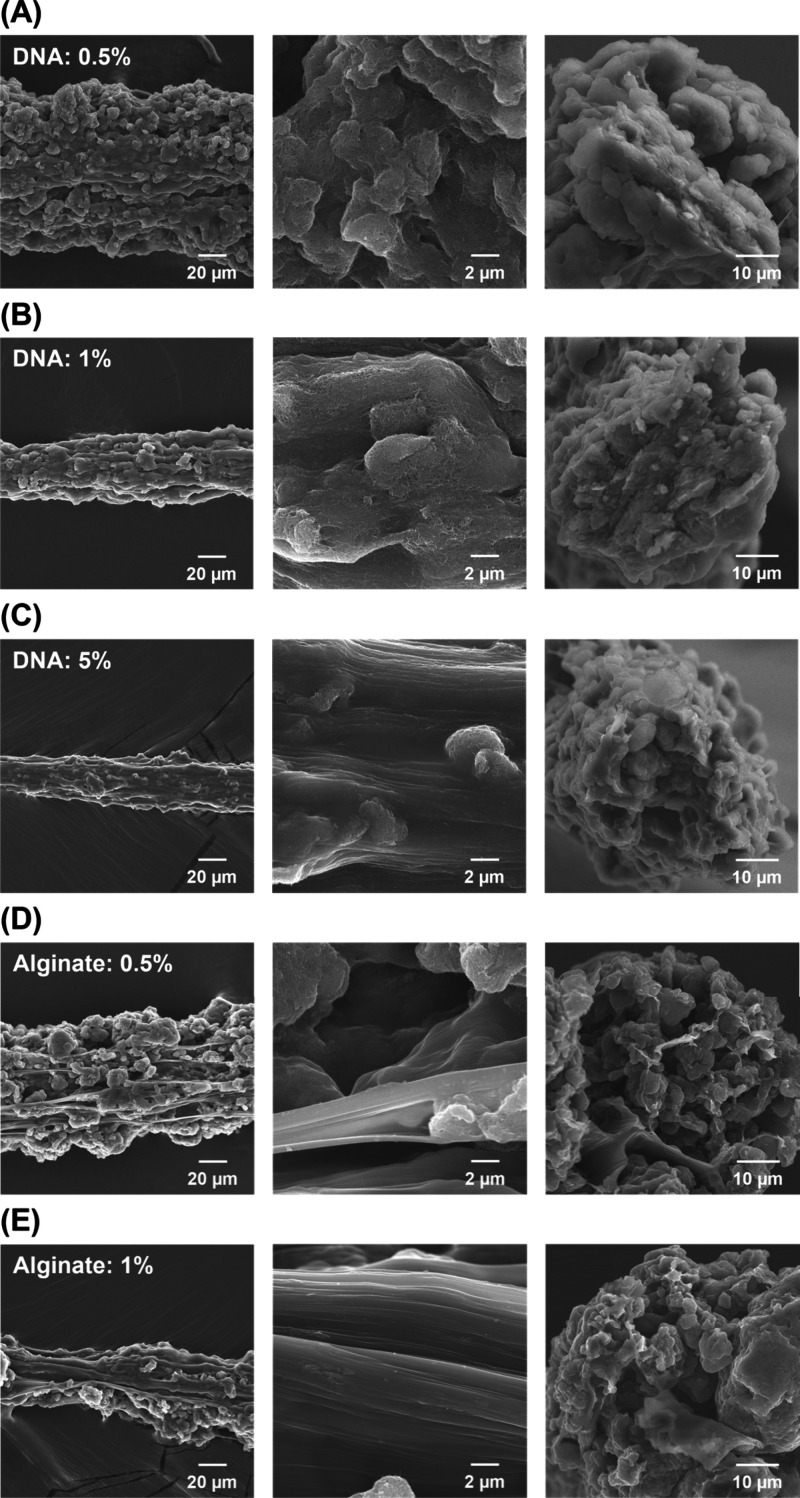
SEM images of the fibers. Chitosan/DNA–CNT fibers with (A) 0.5% (w/v) DNA, (B) 1% (w/v) DNA, and (C) 5% (w/v) DNA. Chitosan/alginate–CNT fibers with (D) 0.5% (w/v) alginate and (E) 1% (w/v) alginate. Low-magnification, high-magnification, and cross-sectional SEM images are shown from left to right, respectively. All fibers were formed with 0.25% (w/v) chitosan and 50 mg/mL CNT.

### Conductivity of Chitosan/DNA–CNT Fibers

The conductivities of the fibers were also investigated. Figure 3A shows a light-emitting diode (LED) illuminated using two dry-cell batteries using a single chitosan/DNA–CNT fiber, indicating that these fibers can be effectively used in electronic applications. Figure 3B shows the resistance of each fiber to different concentrations of DNA or alginate (chitosan: 0.25% (w/v); CNT: 50 mg/mL). If the fiber is uniform, the resistance is inversely proportional to the square of the diameter. In chitosan/DNA–CNT fibers, CNTs interact with the DNA that forms the fiber structure, resulting in a relatively uniform distribution of CNTs. In contrast, chitosan/alginate–CNT fibers possess less homogeneous CNT arrangements, leading to deviations from the ideal inverse trend. The chitosan/DNA–CNT fibers tended to exhibit lower resistance values compared to chitosan/alginate–CNT fibers at the identical diameter. In contrast, the values of conductivity exhibited high variability, with the coefficient of variation reaching 85.6% and 141% for chitosan/DNA–CNT and chitosan/alginate–CNT fibers, respectively. This variability arises from the manual fabrication process, and more significantly, it indicates that the lack of favorable interactions in the chitosan/alginate–CNT fibers leads to the formation of discontinuous CNT networks. It should be noted that alginate differs from DNA not only in CNT affinity but also in molecular structure, viscosity, and interfacial behavior. Therefore, the present comparison between DNA and alginate does not strictly isolate the effect of DNA–CNT interactions alone, and a more systematic comparison using polymers with similar physicochemical properties would be required to clarify the specific contribution of DNA–CNT interactions.

**Figure 3:**
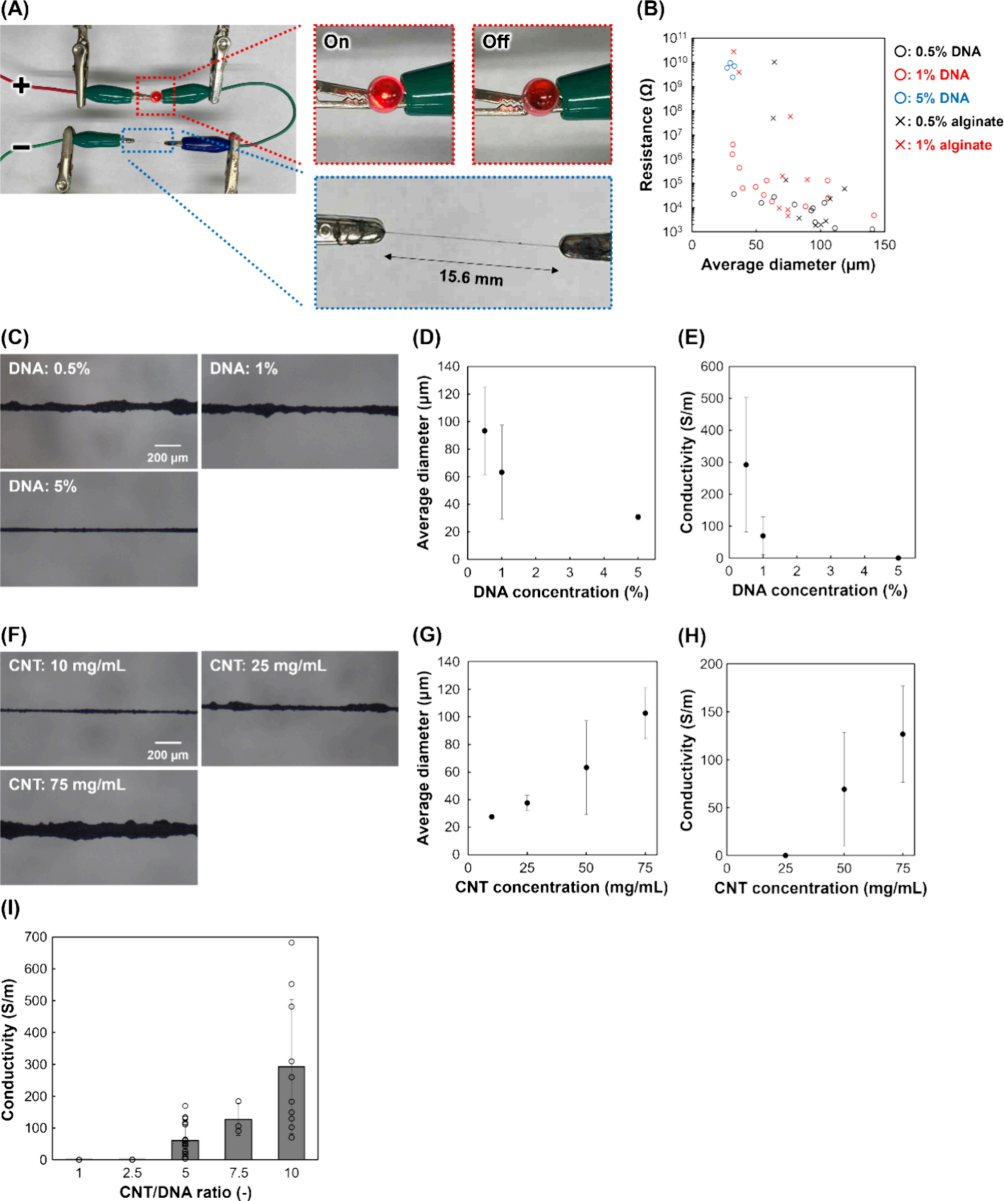
Conductivity of the fibers. (A) Photograph of the LED illumination experimental setup. As a demonstration, an LED was used. Conditions: 0.25% (w/v) chitosan, 1% (w/v) DNA, 50 mg/mL CNTs, and a potential of 3.0 V. (B) Resistance using various concentrations of DNA or alginate. Conditions: 0.25% (w/v) chitosan and 50 mg/mL CNTs. The 5% alginate solution exhibited high viscosity, which hindered CNT dispersion and fiber formation. (C–E) Fibers with various DNA concentrations (0.25% (w/v) chitosan and 50 mg/mL CNTs): (C) images, (D) average diameter (*n* = 6–14), and (E) conductivity (*n* = 4–12). (F–H) Fibers with various CNT concentrations (0.25% (w/v) chitosan and 1% (w/v) DNA): (F) images, (G) average diameter (*n* = 2–14), and (H) conductivity (*n* = 3–12). (I) Relationship between conductivity and CNT/DNA ratio (*n* = 3–18). Error bars represent standard deviations.

Next, the concentrations of the DNA and CNTs on fiber properties were investigated. The diameter and conductivity of the fibers decreased with increasing DNA concentration (Figures 3C–E). Conversely, the diameter and conductivity of the fibers increased with increasing CNT concentration (Figures 3F–H). A greater amount of free CNTs promoted the formation of larger aggregates, resulting in thicker fibers. Accordingly lower DNA (0.5% (w/v)) and higher CNT (75 mg/mL) concentrations led to higher conductivity (100–500 S/m), owing to the increased presence of free CNTs. These results indicate that the CNT/DNA ratio is crucial for conductivity. The conductivity increased when the ratio exceeded 5 (Figure 3I). However, notably, when the DNA concentration is too low, IPC fibers cannot be formed with chitosan. The large variability of diameter and conductivity is attributed to manual fabrication process. In addition, when 1% DNA and 50 mg/mL CNT were used, the variability remained extremely large despite measuring more than ten independent samples. This is likely because the CNT/DNA ratio under this condition allows residual free CNTs to remain and places the system near the percolation threshold, both of which can significantly amplify sample-to-sample variation. Reported conductivities of polymer–CNT composite fibers span several orders of magnitude, such as that of 2,2,6,6-tetramethylpiperidine-1-oxyl-oxidized cellulose nanofibrils-CNTs/poly­(acrylic acid) (2.94 S/m),^27^ polyurea/CNT (18.6 S/m),^28^ and chitosan based CNT/polyurethane (730 S/m).^29^ The conductivity of the chitosan/DNA–CNT fibers was comparable to that of previously reported polymer–CNT composite fibers. In contrast, the conductivity was lower than that of conventional IPC or INC fibers (Table S1). From this comparison, the differences in conductivity may be attributed to the type of conductive nanomaterial used. In this study, 1% (w/v) DNA and 50 mg/mL CNT were used because they exhibited sufficient conductivity while preserving the structure of the thin fibers.

### Applications of Chitosan/DNA–CNT Fibers

Applications of chitosan/DNA–CNT fibers were then demonstrated. Even after being bent to 90°, the dried fibers showed no structural damage (Figure S6A) and retained their conductivity (Figure S6B) indicating their suitability for flexible electronics. As the fibers are composed of hydrogels, they can easily transition between wet and dry states. Notably, the fibers exhibited conductivity even immediately after fabrication in the wet state (Figure S7). Furthermore, the conductivity of the fibers could be restored through simple hydration owing to self-healing, which arises from the reversible electrostatic interactions between chitosan and DNA. The recovery of the conductivity in this fiber via self-healing was therefore evaluated. Figure 4A shows the original, cut, swollen, and self-healed fibers. The cut fibers were self-healed with a 3 mm overlap and successfully rejoined through simple hydration. First, self-healing was confirmed for both chitosan/DNA–CNT and chitosan/alginate–CNT fibers (Figure 4B). For the chitosan/DNA–CNT fibers, the current was not detected immediately after wetting and recovered as drying proceeded (Figure 4B). In contrast, for the chitosan/alginate–CNT fibers as a control, the current recovered immediately after wetting and remained unstable until the fibers dried (Figure 4B). Once dried, the chitosan/DNA–CNT fibers hardly reabsorb water, and conductivity is not restored unless the cut fibers dry and contact each other, indicating that self-healing behavior is likely driven by electrostatic interactions between polymer chains located near the fiber surfaces. In contrast, the chitosan/alginate–CNT fibers likely allow immobilized CNTs to regain mobility, thereby facilitating the rapid formation of conductive pathways. After recovery, the current values became higher than those of the original fibers due to an increase in the contact area caused by overlapping (Figure 4C). The stability of the current during recovery and the variation in the current after recovery can be attributed to interactions between CNTs and the polymer. The use of DNA restricts CNT mobility through DNA–CNT interactions, maintaining the primary conductive pathways and stabilizing the current response. In contrast, CNTs are physically trapped between the chitosan/alginate domains in the fibers, and the conductive pathways between these CNT aggregates can fluctuate, as nonfunctionalized CNTs do not interact with alginate.^30^ As a result, the current is unstable during the healing process, and the current values after healing show large variability. Therefore, DNA–CNT interactions ensure a stable current response both during and after the self-healing process. In this experiment, the fibers were connected by overlapping them; therefore, it is difficult to analyze the conductivity after repeated self-healing cycles. Connection between the cut surfaces and evaluation of repeated self-healing will be addressed in future work. In addition, mechanical characterization of the healed fibers will be investigated in future work.

**Figure 4:**
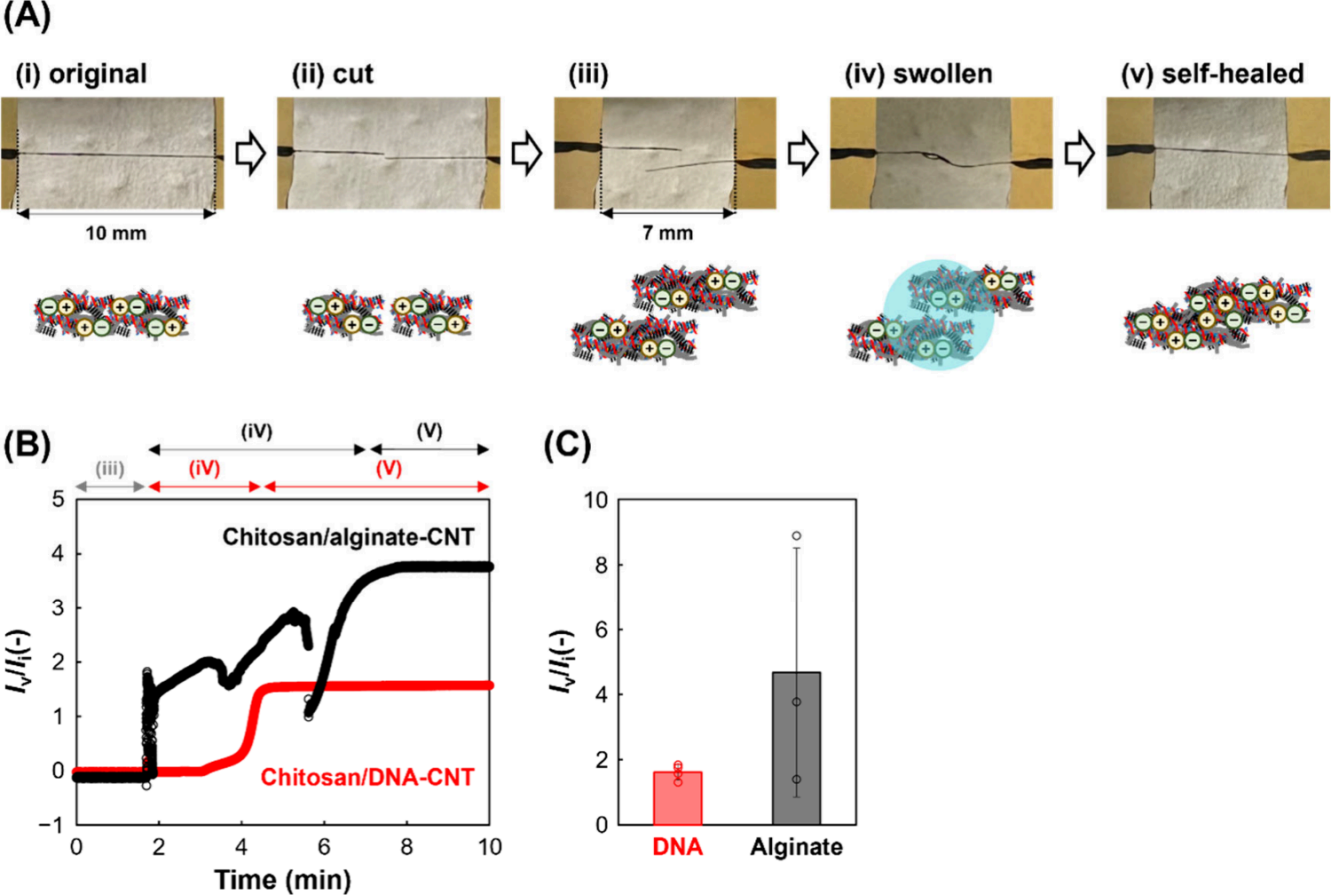
Conductive fiber applications for self-healing. (A) Photographs and illustrations of chitosan/DNA–CNT fibers: (i) original, (ii and iii) cut, (iv) swollen, and (v) self-healed. (B) Amperograms showing the recovery of conductivity during self-healing using DNA or alginate. *I*_i_ and *I*_v_ indicate the current values measured at an applied voltage of 0.5 V across a 10 mm length of the original and self-healed fibers, respectively. Conditions: potential of 0.5 V and 1% (w/v) alginate. (C) Recovered current values normalized to the original current values (*n* = 3–4). Error bars represent standard deviations.

Owing to their stretchability, these fibers are also promising candidates for wearable sensors that detect strain-induced conductivity changes.^21^ Initially, the stretchability of the fibers was evaluated. The dried fibers exhibited no stretchability in air. Subsequently, as-prepared fibers could be stretched up to approximately 55% strain (Figures S8A and C). In addition, the dried fibers could also be stretched up to approximately 40% strain in water (Figures S8B and C). However, once dried, the fibers exhibited limited rehydration capacity and quickly dehydrated upon exposure to air; therefore, the as-prepared fibers were employed for the experiments. Next, the relationship between applied strain and the resulting change in resistance was investigated. The applied strain led to a decrease in current, indicating an increase in resistance (Figure 5A). The greater increase in resistance at higher strain levels suggests the potential for application as a strain sensor (Figure 5B). Finally, the as-prepared fibers were placed on fingers and subjected to stretching through finger motions (Figure 5C). The current values fluctuated with finger stretching (Figure 5D), indicating that the resistance of the fibers changed owing to the mechanical deformation. The obtained relative resistance changes of 40% corresponded to approximately 40% strain based on the calibration curve, which is highly reasonable. The current value rapidly responded to finger bending and straightening and subsequently reached a stable state (Figure S9). Although the dried fiber withstood moderate deformation, such as simple bending (Figure S4), they were unable to endure the intensive stretching induced by finger bending (Figures S10A and B). Conversely, Pt wires retained stable current even during finger bending (Figure S10C). Overall, the fibers exhibited stable conductivity under stretching conditions. Moreover, they demonstrated self-healing function through electrostatic interactions, enabling reversible transitions between dry and wet states, and a strain-sensing capability by exploiting their stretchability in the wet state.

**Figure 5:**
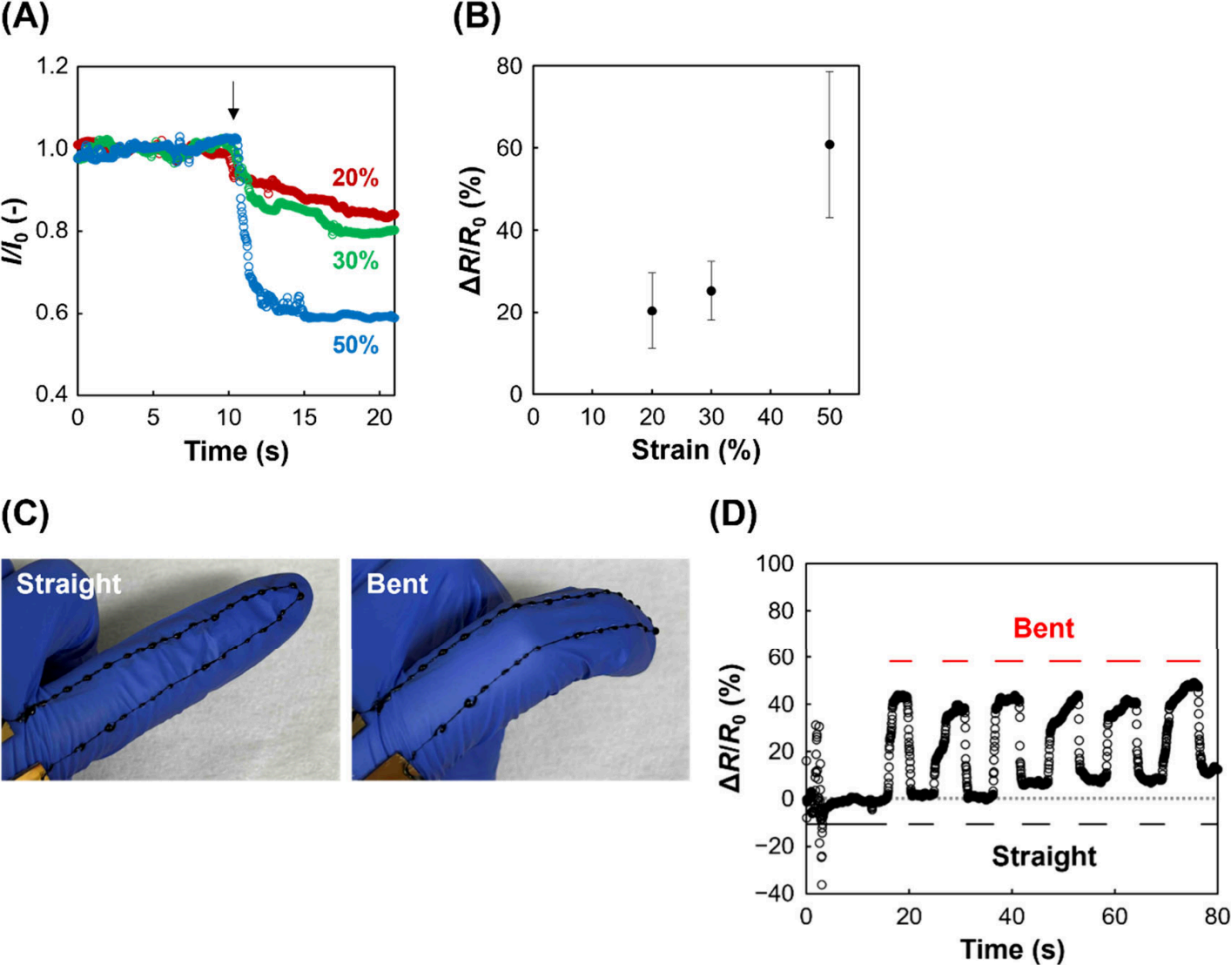
Conductive fiber applications for capturing motion. (A) Current responses to applied strain. The black arrow indicates the application of strain. (B) Relative resistance changes vs strain (*n* = 3). (C) Photographs of wet chitosan/DNA–CNT fibers on a finger during straightening and bending. (D) Relative resistance changes. The potential was 0.5 V. Error bars represent standard deviations.

Next, the effect of DNA cleavage or loss of hybridization on fiber conductivity was investigated. The fibers were treated with 0.1 U/μL DNase I solution (Figure S11A). Chitosan complexes are known to protect DNA from enzymatic degradation by DNases^31^ and previous studies have shown that ssDNA bound to CNTs is resistant to degradation by DNase.^32,33^ Consistently, no significant differences in resistance were observed before and after DNase treatment (Figure S11B). This suggests that the attachment of CNTs to the fiber surface may have inhibited DNA degradation (Figure 2A). Alternatively, the degradation of DNA has little to no impact on conductivity because the CNTs form a conductive network. This result suggests that the chitosan/DNA–CNT fibers retained their conductivity and functionality even in the presence of DNase. Next, the change in conductivity with increasing temperature was monitored because loss of hybridization may lead to changes in conductivity. The temperature increased from room temperature to 100 °C. Mineral oil was used to prevent fibers from drying (Figure S12A). The current increased with temperature in both chitosan/DNA–CNT and chitosan/alginate–CNT fibers. Owing to differences in fiber thickness, the current was normalized to the minimum value. As a result, chitosan/DNA–CNT fibers exhibited a more than 3.4- to 38-fold increase, whereas chitosan/alginate–CNT fibers showed an approximately 1.1- to 3.6-fold increase (Figures S12B and C). An increase in temperature likely led to an improvement in conductivity. Temperature-induced denaturation may contribute to this effect by enabling new interactions with CNTs or by triggering the rearrangement of the CNT network on the fibers. Alternatively, the detachment of DNA from the CNTs due to an increase in temperature may be responsible for this. However, various factors contribute to conductivity, and further investigation is warranted.

Recently, conductive fibers have been increasingly engineered to exhibit multiple functionalities such as temperature sensors.^34^ The IPC process offers the advantage of facile materials incorporation. However, there is a limit to the amount of material that can be incorporated, and incorporating multiple materials often compromises functionality. Therefore, we focused on Janus fibers, which can integrate multiple functions without mixing individual materials. The IPC process enables the fibers to be readily aligned in parallel immediately after fabrication. Janus fibers composed of conductive and magnetic components were fabricated (Figure 6A). The fusion of IPC fibers was driven by the surface tension between the wet fibers.^35^ After drying, the conductive and magnetic parts were separated (Figure 6B), indicating that Janus fibers were successfully fabricated. The SEM image shows that the magnetic fibers contain many magnetic beads (Figure 6C). Because the magnetic beads were functionalized with carboxyl groups, they were extensively retained in the fibers through interactions with chitosan. In addition, the conductive and magnetic fibers were parallel (Figure 6D). The fibers were magnetically attracted when placed approximately 20 mm away from a magnet (diameter: 30 mm; height: 15 mm). Electrical switching was performed using remote magnetic actuation. The electrical circuit was closed by bringing the fibers into contact with the electrode placed 5 mm apart using a magnetic force (Figure 6E). When a conductive/magnetic Janus fiber was used, current was observed as it approached the magnet (Figure 6F). In contrast, no current was observed when the magnetic fiber was used. Thus, electrical switching was achieved via remote magnetic actuation by integrating a magnetic fiber into a conductive fiber. Moreover, 20 consecutive switching cycles were performed to investigate the operational stability and reproducibility of the fiber. The maximum current in each ON state was defined as *I*_max_, and their changes over 20 measurements were examined. The *I*_max_ remained stable throughout the 20 switching cycles (Figure 6G), indicating that the magnetic actuation did not impair conductivity, thereby suggesting that the conductive network and overall fiber structure remained intact. The Janus structure can integrate different functions while maintaining separation, providing a clear advantage. The conductivity of the fibers fabricated by mixing magnetic beads into the same solution as the CNTs was then compared with that of the Janus fibers. The two types of fiber exhibited comparable conductivity (Figure S13A). However, the magnetic beads tend to aggregate in the mixed fibers and might not be distributed throughout the fiber (Figure S13B). Therefore, Janus fibers have the potential to provide uniform functionality across the entire fiber.

**Figure 6:**
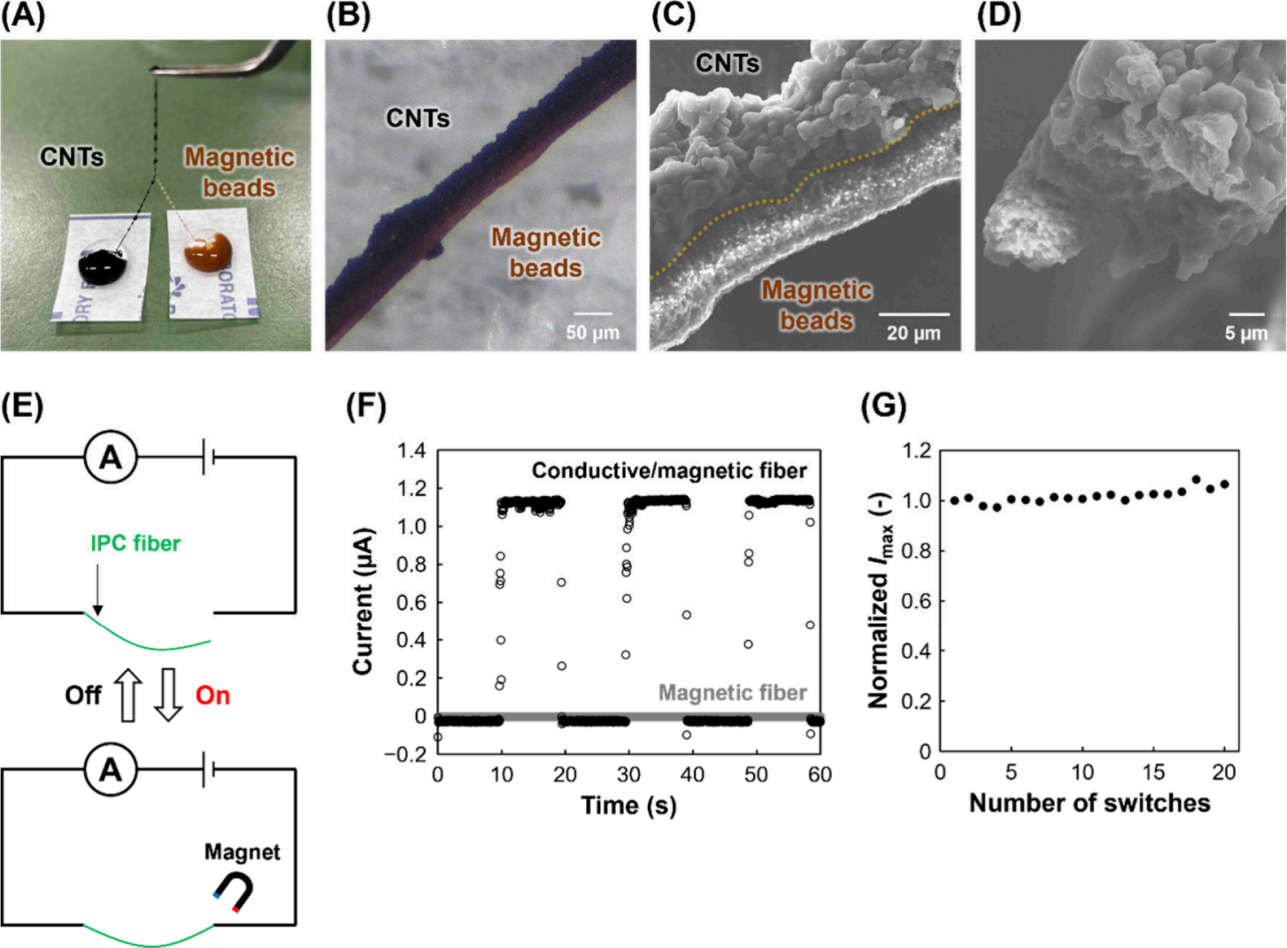
Conductive/magnetic Janus fibers. Photographs of the fiber (A) during the process and (B) after drying. The magnetic fibers were composed of 0.25% (w/v) chitosan, 1% (w/v) DNA, and 2 × 10^9^ beads/mL. SEM images of the (C) surface and (D) cross section of fibers. (E) Schematic illustration of electrical switching via remote magnetic actuation. (F) Amperograms during electrical switching. The potential was 0.5 V. (G) Normalized *I*_max_ for each of the 20 switching cycles.

## Conclusion

In this study, conductive fibers composed of chitosan and DNA–CNT composites were successfully developed using a simple IPC process without any complex processes or specialized equipment. The surface of the chitosan/DNA was covered with CNTs, and the fibers exhibited a densely packed internal structure because of the interaction between DNA and CNTs. In addition, chitosan/DNA–CNT fibers exhibited stable conductivity owing to this interaction compared to chitosan/alginate–CNT fibers. Importantly, the CNT/DNA ratio was found to be a key determinant of the electrical properties of the fibers. The fibers also exhibited flexibility, self-healing capability, and stretchability for strain sensing. The interaction between DNA and CNTs also contributed to the stability of conductivity during and after self-healing. Moreover, the IPC process enables easy fabrication of Janus fibers and the addition of additional functions, such as magnetism. These features render conductive fibers promising candidates for applications in biointerfaced and flexible electronics. On the other hand, further functionalization of conductive fibers leveraging the unique properties of DNA, as well as elucidation of their underlying mechanisms, remains a challenge for future research.

## Supplementary Material



## Notes

CNT dispersibility, fiber length, photograph of a coin placed on a mesh, photographs of the fibers in Milli-Q water, results of Raman spectroscopy, influence of fiber bending, photograph of a self-healed fiber, resistances of wet and dried fibers, changes in current in response to finger bending, capturing of motion using dried fibers, conductivity measurements with DNase treatment, conductivity monitoring with temperature treatment, and comparison between fibers mixed with magnetic beads and Janus fibers (PDF)
