# Supplementary material for: Conductive Fibers of Chitosan/DNA Interfacial Polyelectrolyte Complexation Incorporating Carbon Nanotubes
Source: ACS Appl Mater Interfaces. 2026 Apr 8;18(15):22404–13. doi: 10.1021/acsami.6c00347 (PMC13107379; doi:10.1021/acsami.6c00347)
Supplement: Supplementary file 1 [file am6c00347_si_001.pdf]

Supporting Information

Conductive Fibers of Chitosan/DNA Interfacial Polyelectrolyte Complexation Incorporating Carbon Nanotubes

Yoshinobu Utagawa[1], Masahiro Takinoue[2,3,4], Shin-ichiro M. Nomura[1], Yusuke Sato[5], Hiroaki Onoe[6], Toshinori Fujie[3,4], Hikaru Nakazawa[1], Mitsuo Umetsu[1], Hiroya Abe[1,7], Hitoshi Shiku[1,*], Kosuke Ino[1,**]

[1] Graduate School of Engineering, Tohoku University, Sendai 980-8579, Japan

[2] Department of Computer Science, School of Computing, Institute of Science Tokyo, Yokohama, 226-8501, Japan

[3] School of Life Science and Technology, Institute of Science Tokyo, Yokohama, 226-8501 Japan

[4] Research Center for Autonomous Systems Materialogy, Institute of Integrated Research, Institute of Science Tokyo, Yokohama, 226-8501 Japan

[5] Department of Intelligent and Control Systems, Kyushu Institute of Technology, 680-4 Kawazu, Iizuka, Fukuoka 820-8502, Japan

[6] Department of Mechanical Engineering, Keio University, Hiyoshi, Kohoku-ku, Yokohama 223-8522, Japan

[7] Frontier Research Institute for Interdisciplinary Sciences, Tohoku University, Sendai 980-8578, Japan

*,** Corresponding authors

E-mail address: hitoshi.shiku.c3@tohoku.ac.jp, and kosuke.ino@tohoku.ac.jp

Contents

-Fig S1

-Fig S2

-Fig S3

-Fig S4

-Fig S5

-Fig S6

-Fig S7

-Fig S8

-Fig S9

-Fig S10

-Fig S11

-Fig S12

-Fig S13

-Table S1

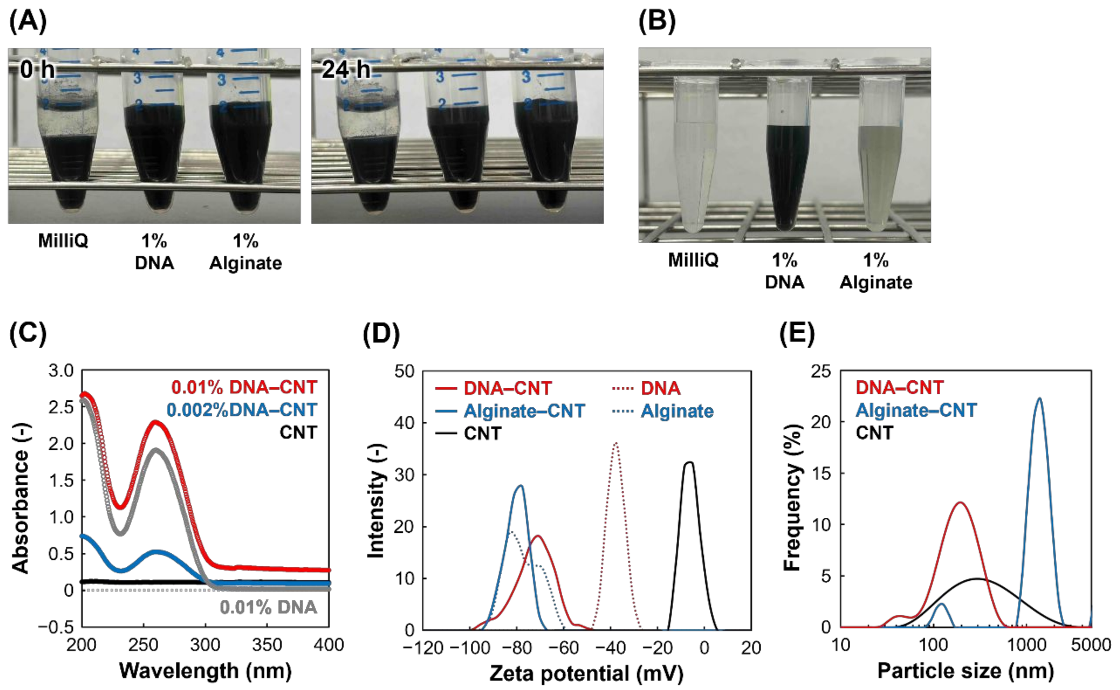

Figure S1

CNT dispersibility. (A) Photographs of CNT dispersion using milli-Q, 1% (w/v) DNA solution, and 1% (w/v) alginate solution after 0 and 24 h from sonication. (B) Photographs of supernatants after centrifugation (2500 × g, 30 min). (C) UV-Vis spectra of DNA–CNT composite suspension. Initially, 0, 0.2, and 1% (w/v) DNA solutions containing 1 mg/mL CNT were prepared and sonicated. Subsequently, supernatants were prepared by centrifugation. The supernatants were diluted 10-fold and analyzed. (D) Zeta potentials. (E) Particle sizes. The supernatants in (B) were diluted 100-fold with Milli-Q water and used for zeta potential and particle size measurements.

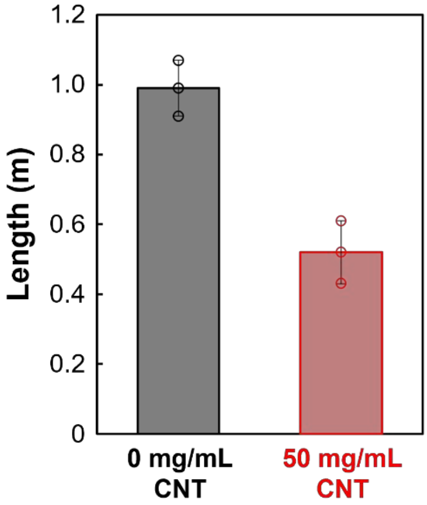

Figure S2

Fiber length using a DNA droplet containing 0 or 50 mg/mL CNTs (n = 3). Chitosan: 0.25% (w/v); DNA: 1% (w/v). Error bars represent standard deviations.

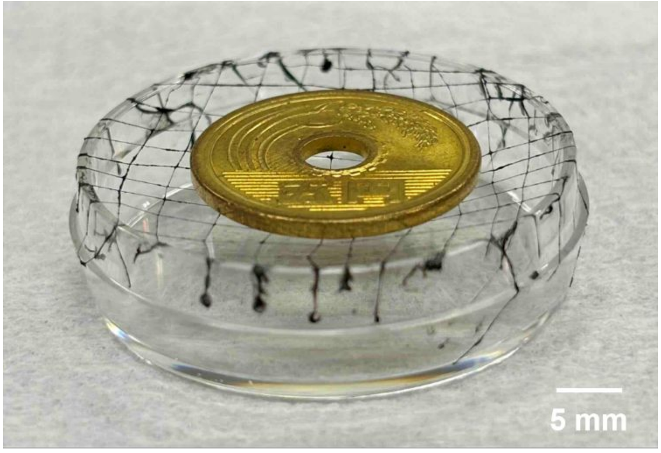

Figure S3

Photograph of a coin placed on a mesh made of chitosan/DNA–CNT fibers. The weight of the coin is 3.75 g.

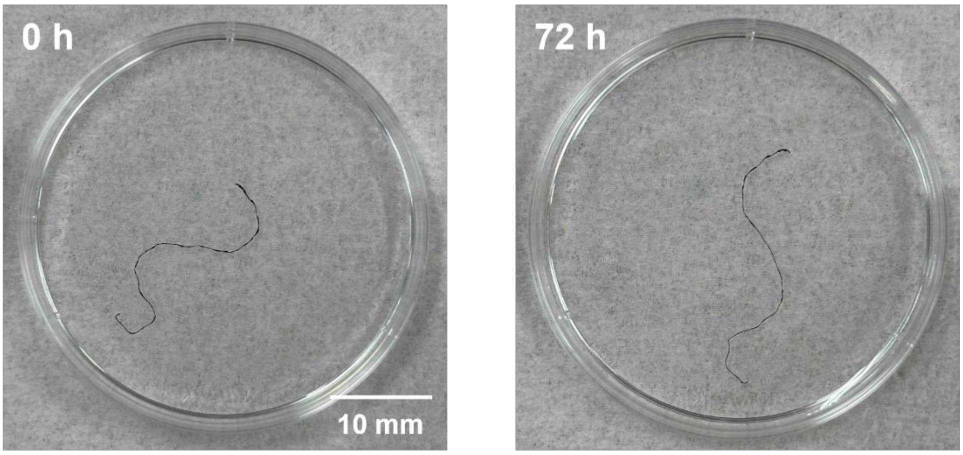

Figure S4

Photographs of chitosan/DNA–CNT fibers in Milli-Q water after (A) 0 and (B) 72 h of incubation. Chitosan: 0.25% (w/v); DNA: 1% (w/v); CNT: 50 mg/mL.

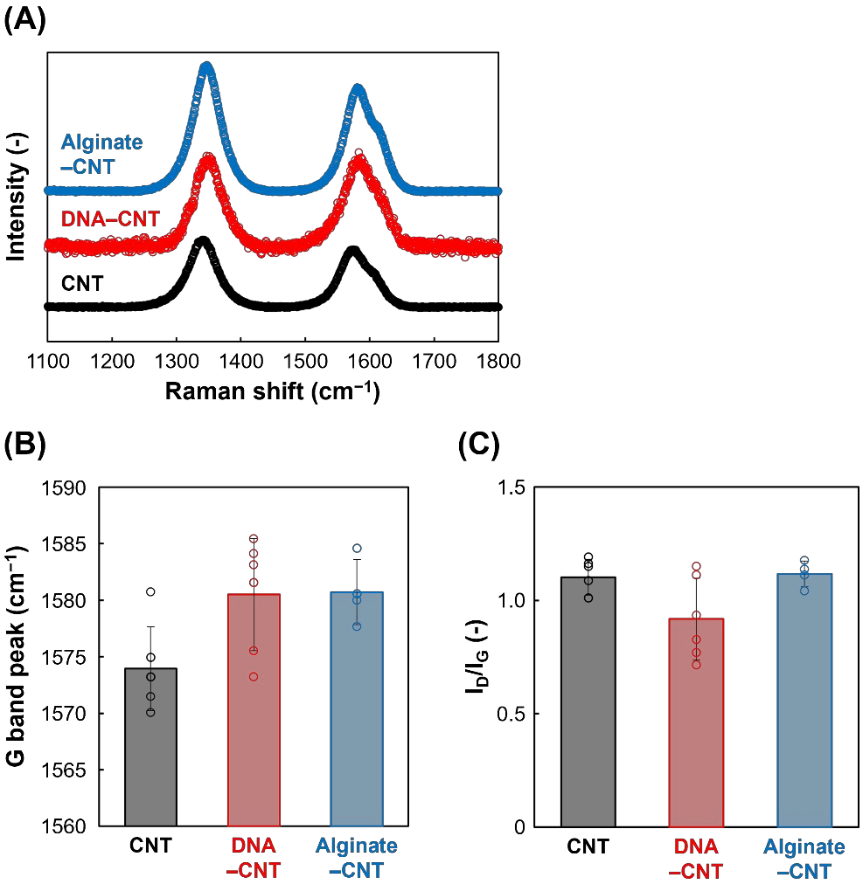

Figure S5

Raman spectra of CNTs. (A) Spectra of CNT, chitosan/DNA–CNT fibers, and chitosan/alginate–CNT fibers. (B) G band peaks (n = 4–6). (C) Intensity ratio of the D band to G band (I_D/I_G) obtained from Raman spectra (n = 4–6). Error bars represent standard deviations.

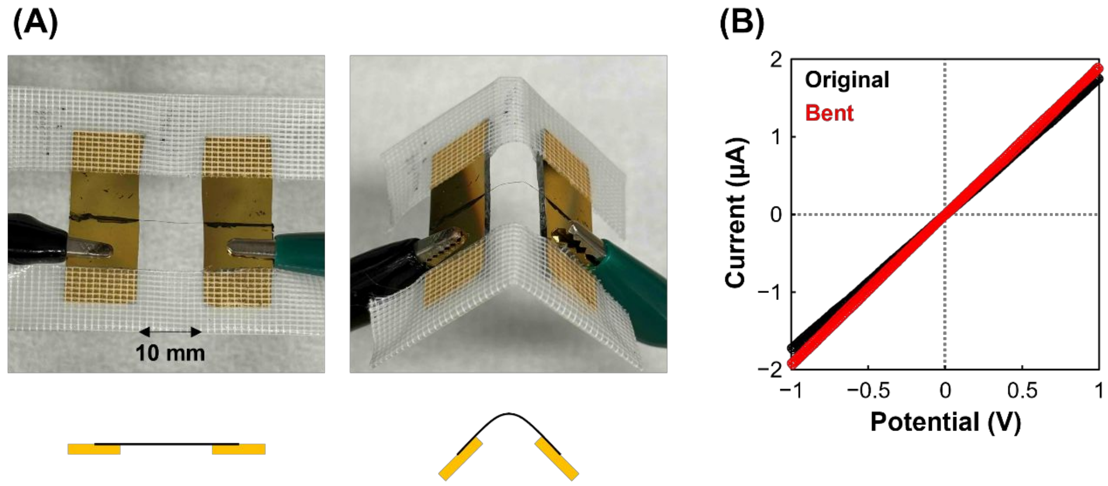

Figure S6

Influence of fiber bending on the electrical performance of the chitosan/DNA–CNT fibers. (A) Photograph of the fibers in the straight and bent states. (B) Current–voltage measurements.

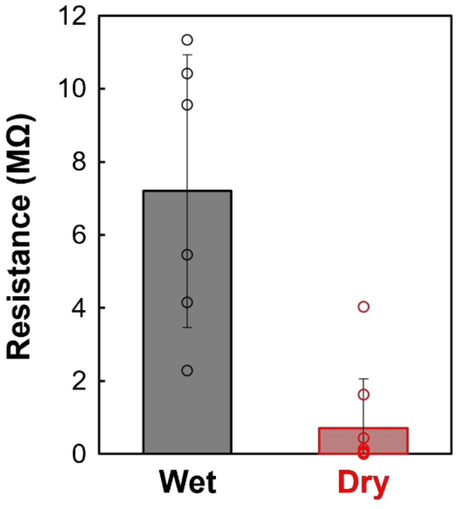

Figure S7

Resistances of wet and dried chitosan/DNA–CNT fibers (*n* = 6–9). Error bars represent standard deviations.

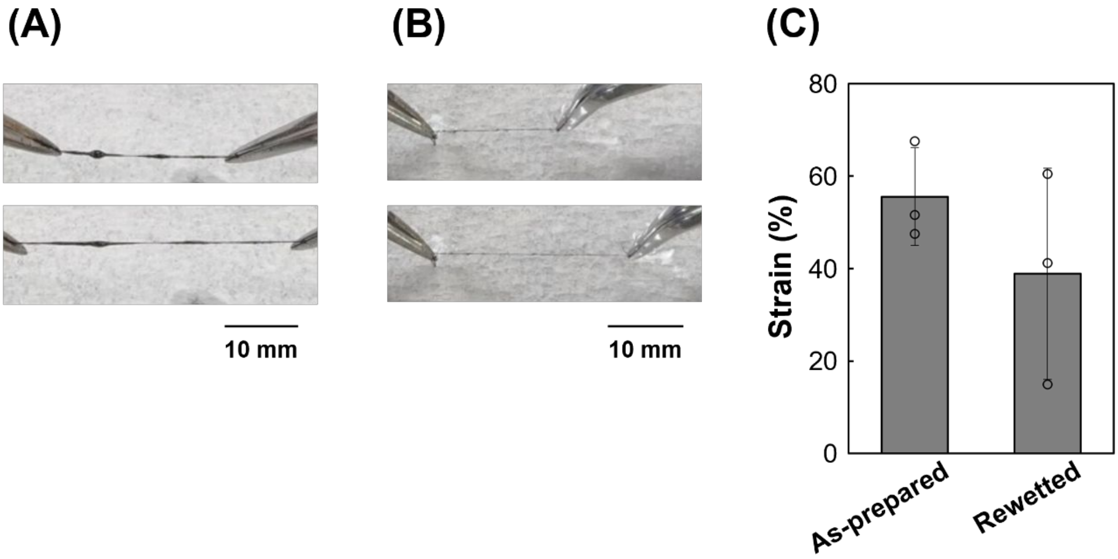

Figure S8

Stretchability of the fibers. (A, B) Photographs of (A) as-prepared and (B) rewetted fibers. (C) Strain of each condition (n = 3). Error bars represent standard deviations.

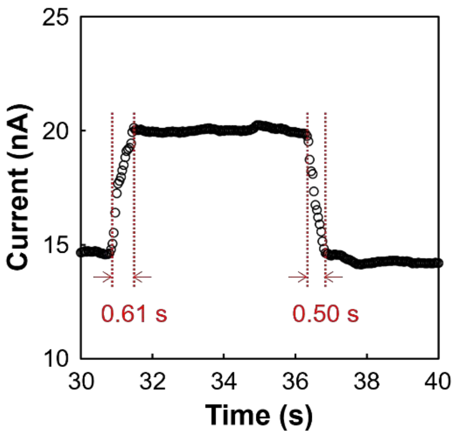

Figure S9

Current changes responded to finger bending and straightening using chitosan/DNA–CNT fibers.

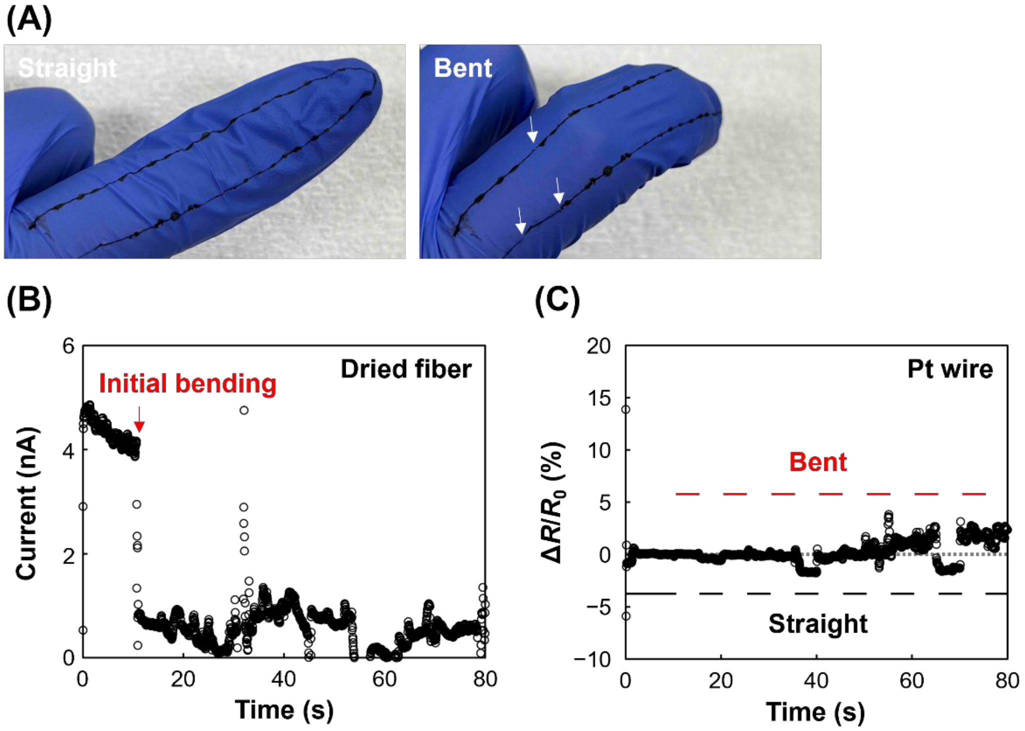

Figure S10

Dried chitosan/DNA–CNT fiber and Pt wire for motion capturing. (A) Photograph of a dried chitosan/DNA–CNT fiber on a finger during bending and straightening. White arrows indicate disconnections. (B) Amperogram of the fiber using under the dried chitosan/DNA–CNT fiber. Potential: 0.5 V. (C) Relative resistance changes using Pt wire. Potential: 0.1 V.

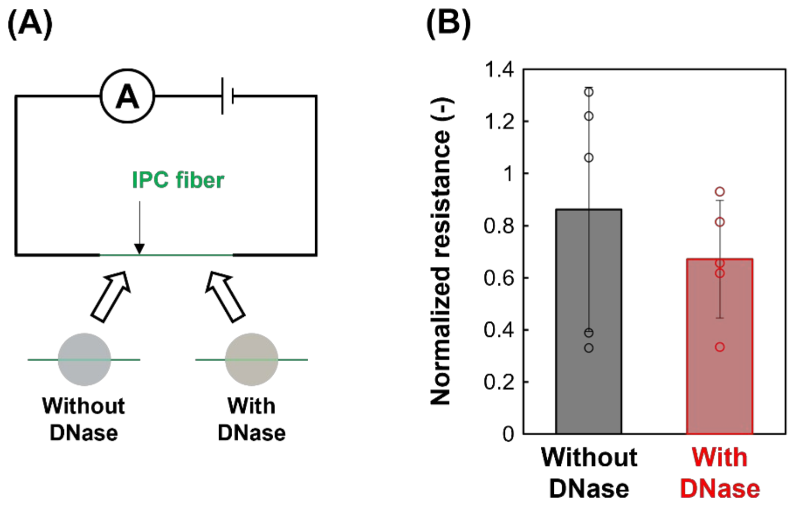

Figure S11

Conductivity measurements with DNase treatment. (A) Schematic. The fibers were treated with a Tris-HCl buffer (pH 8) containing 0.1 U/μL DNase and 0.5 mM $MgSO_4$ for 30 min. (B) Normalized current without or with DNase (n = 5). The normalized resistance was calculated by dividing the resistance after treatment by the resistance before treatment. Error bars represent standard deviations.

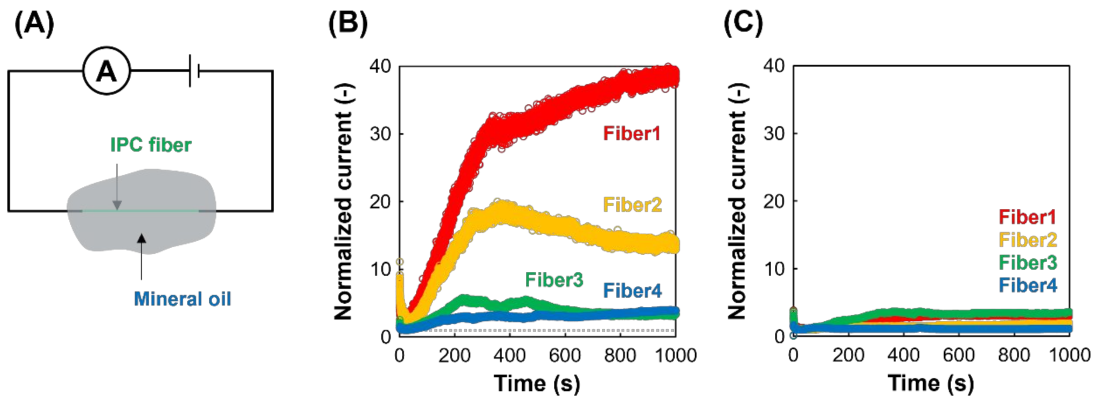

Figure S12

Conductivity monitoring with temperature treatment. (A) Schematic. The wet fibers were sealed with polyimide tape, and mineral oil was applied to the sealed fibers to prevent the fibers from drying. Normalized currents using (B) chitosan/DNA–CNT and (C) chitosan/alginate–CNT fibers.

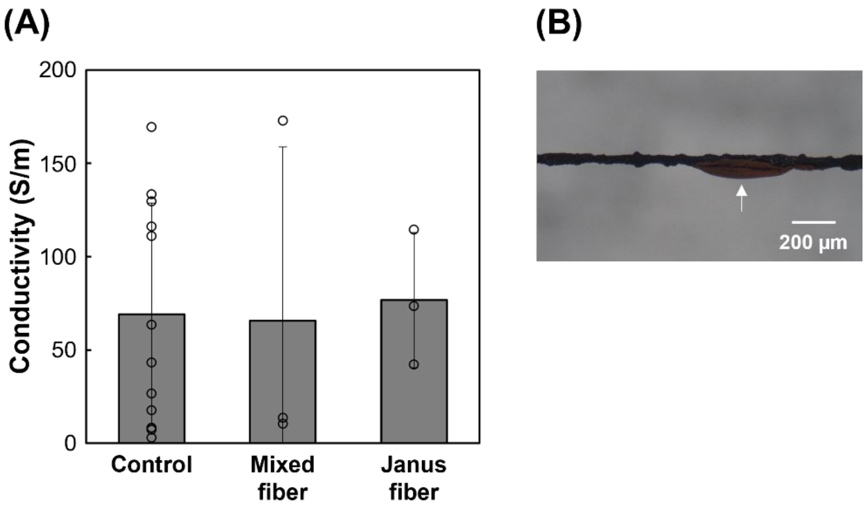

Figure S13

Comparison between fibers mixed with magnetic beads and Janus fibers. (A) Conductivity (n = 3–12). (B) Image of the fibers mixed with magnetic beads. White arrows indicate magnetic beads that are aggregated. Error bars represent standard deviations.

Table S1 Conductivity of IPC and INC fibers

Materials	Conductivity (S/m)	Ref.
Chitosan/cellulose nanofibrils–reduced graphene oxide (rGO)	5538.7	1
Aminoguanidine hydrochloride-modified cellulose nanocrystal (AH-CNC)/2,2,6,6-tetramethylpiperidinyl-1-oxy radical oxidized cellulose nanofibers (TO-CNF)–carboxylated multiwalled CNTs	392	2
Chitin nanocrystals/TO-CNF–single walled CNTs	2056	3
AH-CNC/rGO	3298	4

References

1. Lin, Y.; Wen, S.; Peng, X.; Cai, Y.; Geng, L.; Chen, B. Interfacial Polyelectrolyte Complexation Spinning of Graphene/Cellulose Nanofibrils for Fiber-Shaped Electrodes. *J. Mater. Res.* **2020**, *35* (2), 122-131.
2. Zhang, K.; Hujaya, S. D.; Järvinen, T.; Li, P.; Kauhanen, T.; Tejesvi, M. V.; Kordas, K.; Liimatainen, H. Interfacial Nanoparticle Complexation of Oppositely Charged Nanocelluloses into Functional Filaments with Conductive, Drug Release, or Antimicrobial Property. *ACS Appl. Mater. & Interfaces* **2020**, *12* (1), 1765-1774.
3. Zhang, K.; Ketterle, L.; Järvinen, T.; Hong, S.; Liimatainen, H. Conductive Hybrid Filaments of Carbon Nanotubes, Chitin Nanocrystals and Cellulose Nanofibers Formed by Interfacial Nanoparticle Complexation. *Mater. Des.* **2020**, *191*, 108594.
4. Zhang, K.; Ketterle, L.; Järvinen, T.; Lorite, G. S.; Hong, S.; Liimatainen, H. Self-assembly of graphene oxide and cellulose nanocrystals into continuous filament via interfacial nanoparticle complexation. *Mater. Des.* **2020**, *193*, 108791.
